# Metagenomic profiling of the insect-specific virome in non-urban mosquitoes (Culicidae: Culicinae) from Colombia’s Northern inter-Andean valleys

**DOI:** 10.1371/journal.pone.0331552

**Published:** 2025-09-03

**Authors:** Andrés Gómez-Palacio, Howard Junca, Rafael J. Vivero-Gomez, Juan Suaza, Claudia X. Moreno-Herrera, Gloria Cadavid-Restrepo, Dietmar H. Pieper, Sandra Uribe

**Affiliations:** 1 Laboratorio de Investigación en Genética Evolutiva – LIGE, Universidad Pedagógica y Tecnológica de Colombia, Tunja, Boyacá, Colombia; 2 RG Microbial Ecology: Metabolism, Genomics & Evolution, Div. Ecogenomics & Holobionts, Microbiomas Foundation, LT11A, Chia, Colombia; 3 Microbial Interactions and Processes Research Group (MINP), Helmholtz Centre for Infection Research, Braunschweig, Germany; 4 Grupo Microbiodiversidad y Bioprospección, Departamento de Biociencias, Facultad de Ciencias, Universidad Nacional de Colombia, Medellín, Colombia; 5 Grupo de Investigación en Sistemática Molecular (GSM), Departamento de Biociencias, Facultad de Ciencias, Universidad Nacional de Colombia, Medellín, Colombia; Instituto Nacional de Salud Publica, MEXICO

## Abstract

Hematophagous mosquitoes are major vectors of diverse pathogens and serve as bioindicators in tropical ecosystems, yet their virome in non-urban Neotropical regions remains poorly characterized. We analyzed the virome of 147 mosquitoes from two natural ecosystems in Colombia using a hybrid viral identification approach, combining high-confidence and less stringent methods. Most high-confidence viral contigs remained unclassified or unknown, as expected for metagenomic surveys in novel ecosystems. However, members for the Magrovirales and Ortervirales, and other six orders were detected at lower abundance. Using a complementary, less stringent approach, we identified 168 viral species from 68 genera and 22 families across four mosquito tribes (Aedini, Culicini, Orthopodomyiini, Sabethini), with dominance of Metaviridae, Retroviridae, Iridoviridae, and Poxviridae, though many sequences could not be taxonomically assigned. Insect-specific viruses predominated, while no medically relevant arboviruses were detected. Both methods consistently identified Trichoplusia ni TED virus, Cladosporium fulvum T-1 virus, Lymphocystis disease viruses, and Oryctes rhinoceros nudivirus among the most abundant and frequently detected taxa across samples. Alpha diversity indices revealed the highest virome diversity in Sabethini, followed by Orthopodmyiini, and substantially lower richness and diversity in Aedini and Culicini. These results provide a baseline for virome characterization in sylvatic mosquitoes from Colombia and highlight the need for further research on the ecological roles of the mosquito virome in pathogen transmission and microbiome evolution.

## Introduction

Mosquitoes from the Culicidae family, known for their blood-feeding habits, serve as prominent vectors worldwide, transmitting pathogenic viruses, including arthropod-borne viruses (arboviruses), to both humans and wild animals. Notably, mosquito genera such as *Aedes*, *Anopheles*, and *Culex* include species that are recognized as primary vectors for endemic arboviruses, including dengue virus (DENV), yellow fever virus, Zika virus (ZIKV), O’nyong-nyong virus, and West Nile virus [1–5]. In addition to arboviruses, the mosquito virome includes insect-specific viruses (ISVs) and viruses associated with the microbiota, such as bacteriophages and fungi-associated viruses. These viruses play crucial roles in diverse biological processes, including vector competence, as well as in environmental dynamics and evolution [1,6,7]. While insect-specific viruses (ISVs) do not replicate in vertebrates, some share phylogenetic similarities with pathogenic arboviruses. Consequently, there is a growing interest in uncovering and characterizing ISVs, mostly from potential vector species such as *Culex* sp. [8,9]. Also, ISV are explored as models for virus restriction mechanisms or as potential biocontrol agents [1,10,11].

Several studies have shown that the endogenous microbiota can influence the vector competence of *Aedes aegypti* for transmitting arboviruses [12–16]. The discovery of viruses that infect and manipulate the mosquito microbiota is also steadily expanding [17,18]. As an example, ISVs, like Phasi Charoen-like virus (PCLV) and Humaita-Tubiacanga virus (HTV), may not only impact the arbovirus infection capacity but also influence transmission dynamics [19]. Recently, a study on factors controlling dengue virus transmission revealed that gut colonization by Rosenbergiella_YN46 enables both *Ae. albopictus* and *Ae. aegypti* mosquitoes to resist DENV and ZIKV infections [20]. Additionally, Rosenbergiella_YN46 demonstrated effective transstadial transmission in field mosquitoes, blocking the transmission of Dengue Virus serotype 2 (DENV2) by newly emerged adult mosquitoes [20].

In recent years, the virome of mosquitoes has been subjected to increasingly detailed analysis through next-generation sequencing-based (NGS) approaches. However, its precise role in mosquito physiology and species evolution is still undergoing investigation [18]. Recent virome analyses of populations of *Ae. aegypti* and *Ae. albopictus* has revealed significant differences in the composition and diversity of insect-specific viruses (ISVs) present in these mosquito species. Additionally, a comprehensive global analysis of Culicidae family viromes, spanning 128 species across 14 genera within the Anophelinae subfamily and Aedini, Culicini, and Sabethini Tribes, has highlighted the abundance of unclassified viruses [18]. This study has also elucidated the variability in viromes among mosquito species, populations, and even individual mosquitoes, reflecting localized dynamics of viral transmission and environmental factors [18].

The limited data on Neotropical mosquito viromes, particularly in Colombia, emphasizes the urgent need for additional studies on virome composition in regions with high prevalence of vector-borne viral infections [7]. A preliminary metagenomic analysis of mosquitoes from the genera *Ochlerotatus, Culex, Limatus, Mansonia, Psorophora*, and *Sabethes* within a rural savanna ecosystem in the Colombian Orinoco, revealed the dominance of ISVs, primarily from the *Iflaviridae* family, across all mosquito samples [21]. The presence of ISVs in these samples suggests their significant role in local mosquito populations and potential implications for arbovirus transmission [21].

It is estimated that 324 species belonging to the Culicinae subfamily are distributed in Colombia (WRBU, 2017), with particularly high abundance within the Tribes Culicini, Aedini, and Sabethini [22]. Several of these species have been implicated in the transmission of arboviruses from the genera Alphavirus (such as Venezuelan Equine Encephalitis, Eastern Equine Encephalitis (EEEV), and Mayaro), Flavivirus (including Dengue, Yellow fever, West Nile (WNV), and Saint Louis Encephalitis (SLEV)), and Orthobunyavirus (such as Guaroa and Wyeomyia) [23,24]. Despite significant efforts to assess Culicinae taxonomy in various regions of Colombia [25–28], studies on mosquito systematics and diversity are still needed, particularly in non-described or underrepresented native foci biotopes such as the inter-Andean valley forests of Northern Colombia.

In addition to PCR-based identification studies of arboviruses in *Ae. aegypti* and *Ae. albopictus*, investigations in Colombia have targeted *Culex sp.* [29–32]. To date, only few studies utilizing NGS-based approaches in wild-caught mosquitoes to assess virome composition and diversity are available [7,21,33,34]. Furthermore, the complexities of mosquito systematics, driven by high species diversity and cryptic species complexes within the Culicidae family, pose challenges in accurately identifying the nature of the virome associated with wild-caught mosquitoes in Colombia’s natural non-urban ecosystems.

The virome characterization in the urban sympatric species *Ae. aegypti* and *Ae. albopictus* from Colombia indicated that viromes differ strikingly between species in abundance and diversity [33], and the ISVs abundance is influenced by arboviruses such as dengue virus [21], whereas in non-urban species from the eastern plains it was primarily shaped by ISVs such as the most abundant Hanko iflavirus-1 in *Culex eknomios* and *Ochlerotatus serratus*, and other Negevirus genus are common viral species among the mosquitoes, although in lower proportions [21]. Overall, the mosquito virome is shaped by local conditions, including mosquito species and habitat, as well as by environmental factors such as food source preferences [33,35,36]. To uncover novel insights into Culicinae diversity and its associated virome, we employed an metataxonomic approach to assess the virome composition and diversity of mosquitoes collected from two localities in the inter-Andean valleys of Northern Colombia. To our knowledge, this study represents the first investigation of viromes in non-urban mosquitoes in Colombia.

## Materials and methods

### Mosquito sampling, RNA isolation, and sequencing

A total of 147 adult mosquitoes were captured using both active methods (mouth aspirators and entomological net) and traps (CDC white LED light trap and Shannon light trap, Center for Disease Control and Prevention, USA) in the rainforest of the Rio Claro River basin in the Puerto Triunfo locality (the middle basin of the Magdalena River), and in the high mountain dry forest in Santa Fe de Antioquia (Table 1**).** In the Rio Claro River basin, sampling was conducted from November 20^th^ to 22^nd^, 2020, in a protected area of the Cañon del Rio Claro, located at 300 meters above sea level (m.a.s.l.) in the northern Inter-Andean Valleys of the Central Cordillera. This area is managed to conserve its natural environment and biodiversity. In Santa Fe de Antioquia, sampling was conducted from December 2nd to 4^th^, 2020, in a semi-rural area on the outskirts of the municipality, located at 510 m.a.s.l. on the border of the Cotové River, characterized by sparsely scattered rural dwellings. The captured mosquitoes were individually stored in Eppendorf tubes containing 1 ml of RNAlater® (Sigma-Aldrich, St. Louis, USA). They were then transported in a portable cooler at 4°C to maintain the temperature and prevent nuclei acid degradation. Including fed females in the analysis could introduce bias by incorporating viral material from the blood meal rather than the mosquito’s native virome. To minimize this potential bias, we used traps designed to target host-seeking (unfed) females, and carefully examined all samples under a stereoscope to determine their feeding status prior to identification. This process ensured that fed females were excluded from the analysis. Once in the laboratory, the mosquitoes were identified using available morphological keys on a frozen surface to ensure accurate identification and RNA preservation [37]. Subsequently, they were sorted according to species/morphotype. For subsequent metagenome analysis, the mosquitoes were grouped into 21 pools (Table 1). Collection of mosquito specimens was conducted in a private property with the correspondent permission of landowners, and under the Colombian Decree N° 1376 of 2013 of the Ministry of Environment and Sustainable Development (MADS).

**Table 1:** Tribe, sample code, number of mosquitoes, origin, and species/morphotype of mosquitoes analyzed in this study.

Tribe	Sample name	No. Of mosquitoes	Locality	Coordinates	Species/ morphotype
**Aedini**	Ad_m08	4	Puerto Triunfo	5° 53’ 25“ N 74° 51’ 30” W	*Ochlerotatus* sp. 1
	Ad_m09	4			*Ochlerotatus* sp. 2
	Ad_m18	3	Santa Fe de Antioquia	6° 31’ 49“ N 75° 49’ 40” W	*Aedes albopictus*
	Ad_m20	9			
	Ad_m21	6		6° 32’ 16“ N 75° 49’ 51” W	*Aedes* sp.
**Culicini**	Cx_m01	10	Puerto Triunfo	5° 53’ 50“ N 74° 51’ 34” W	*Culex* sp. 1
	Cx_m02	12			*Culex* sp. 1
	Cx_m13	10			*Culex* sp. 3
	Cx_m14	5		5° 52’ 56“ N 74° 51’ 28” W	*Culex* sp. 4
	Cx_m15	4			*Culex* sp. 5
	Cx_m17	18	Santa Fe de Antioquia	6° 33’ 51“ N 75° 49’ 48” W	*Culex* sp. 1
	Cx_m24	5	Puerto Triunfo	5° 53’ 38“ N 74° 51’ 46” W	*Culex* sp.
	Cx_m25	5			*Culex* sp.
	Cx_m26	6			*Culex* sp.
**Orthopodmyiini**	Or_m22	5	Santa Fe de Antioquia	6° 32’ 16“ N 75° 49’ 51” W	*Orthopodomyia* sp.1
	Or_m23	7			*Orthopodomyia* sp.2
**Sabethini**	Sa_m05	10	Puerto Triunfo	5° 53’ 25“ N 74° 51’ 30” W	*Wyeomyia* sp. 1
	Sa_m06	3			*Sabethes* sp.
	Sa_m07	4			*Trichoprosopon* sp.
	Sa_m10	8			*Wyeomyia* sp. 2
	Sa_m11	9			*Wyeomyia* sp. 3

Total RNA was extracted using the High Pure Viral Nucleic Acid Kit (Roche Diagnostics, Mannheim, Germany) following the manufacturer’s instructions. To eliminate contaminating DNA, extracted nucleic acids were treated with DNase I (Thermo Fisher Scientific) according to the manufacturer’s protocol. RNA quality was assessed using an Agilent 2100 Bioanalyzer (Agilent Technologies), and only samples with an RNA integrity number (RIN) > 7 were processed further. All RNA samples had concentrations exceeding 100 ng/µL, ensuring sufficient input material for library preparation and sequencing. Libraries were prepared using the TruSeq Total RNA Library Preparation Kit (Illumina) with random hexamer priming, and sequenced on a NovaSeq 6000 S4 system (Illumina) with a 150-bp paired-end protocol at the Helmholtz Centre for Infection Research (HZI, Braunschweig, Germany). Each library was sequenced to an average depth of 17.7 million reads per sample, providing robust coverage for virome analysis.

### Raw data processing

The paired-end reads were processed using the fastp software v.0.20.0 to remove adaptor sequences and perform quality-based filtering [38]. Ribosomal RNA sequences were extracted from the resulting high-quality reads (Phred quality score > 20) using non-redundant sequences from Bacteria, Archaea, and Eukarya available in the SILVA 138.1 database [39]. Ribosomal sequence sorting was conducted using an e-value cutoff of 10^−5^ in SortMeRNA v4.3.4 [40]. Unmapped reads were mapped against the publicly available dataset of the latest reference genomes of *Culex quinquefasciatus* strain JHB (RefSeq: GCF_015732765.1), *Cx. pipiens pallens* (RefSeq: GCF_016801865.2), *Aedes aegypti* strain LVP_AGWG (RefSeq: GCF_002204515.2) and *Ae. albopictus* (RefSeq: GCF_006496715.1) using BWA-MEM v.0.7.17 [41,42].

### Viral Sequence Identification and Taxonomic Annotation

Virome in each pool were analyzed in metagenomes depleted from mosquito-derived reads. The remaining reads were assembled using Metaviral SPAdes v.3.15.5 [43]. To comprehensively identify viral sequences, we employed a hybrid pipeline that integrates both marker-based and deep learning approaches. Assembled contigs ≥1000 bp were analyzed using geNomad [44], in end-to-end mode, which includes viral detection, marker gene classification, neural network prediction, and genome quality estimation via CheckV [45], to ensure robust taxonomic assignment, potential proviruses or Endogenous Viral Elements (EVEs), and completeness assessment. To increase sensitivity to novel, highly divergent, or partial viral sequences that may evade reference-based detection on highly fragmented metagenome assemblies, contigs between 500 bp and 999 bp were analyzed with DeepVirFinder (DVF) [46]. DVF-predicted viral contigs were subsequently validated with geNomad’s neural network classification (*nn-classification* module). For both geNomad-predicted viral contigs ≥1000 bp lacking taxonomic assignment (“Unclassified/Unknown”) and putative viral contigs 500–999 bp identified by DVF, we performed BLASTx [47] searches against the latest NCBI RefSeq viral protein database (viral.1.protein.faa, 2025-05-05) to assign taxonomy and confirm viral origin. For each contig, only the top BLAST hit with the highest score, lowest e-value, and identity >30% was retained. Viral taxonomy nomenclature was confirmed using the International Committee on Taxonomy of Viruses (ICTV - https://ictv.global/taxonomy) 2025 release. This hybrid workflow allowed us to detect both high-confidence and potentially novel viral sequences, balancing discovery with specificity. We assessed the extent to which mosquito tribe explained virome composition by analyzing beta diversity using Bray–Curtis dissimilarity calculated on Hellinger-transformed abundance data. Patterns were visualized via non-metric multidimensional scaling (NMDS) with the metaMDS function from the vegan R package [48]. Group centroids and 95% confidence ellipses were plotted, and tribe effects were tested with PERMANOVA (Adonis, 999 permutations). Differences in alpha diversity indices among mosquito tribes were assessed using the Kruskal–Wallis test, followed by Dunn’s post-hoc comparisons with Benjamini–Hochberg correction for multiple testing [49].

## Results

### Mosquito virome characterization based on strict taxonomic assignment

After sequencing, the raw reads (pre-filtered) were obtained as properly paired forward and reverse reads. These reads were then processed to remove low-quality sequences and host contamination, resulting in a final set of high-quality reads (post-filtered) used for downstream analysis (S1 Table). Mosquito pool samples yielded between 7.8 × 10⁶ and 4.7 × 10⁷ raw reads per sample, with an average of 93.2% (±8.9%) retained after quality control (S1 Table**).** Assembly yielded between 576 and 166,712 contigs per pool (median: 24,498; mean length: 514 ± 227 bp; S1 Table). Three patterns emerged: (i) Culicini showed marked variability, with Cx_m24 producing the highest number of contigs (166,712) despite only moderate sequencing depth, while Cx_m13 exhibited the lowest read retention (52.2%); (ii) Orthopodmyiini samples (Or_m22, Or_m23) generated consistently high numbers of contigs (~105,000) with highly similar lengths (average 453 ± 3 bp); and (iii) within Aedini, Ad_m18 and Ad_m20 produced very few contigs (939 and 576, respectively), while Ad_m08 and Ad_m09 had much higher yields (24,498 and 29,228**).** Non-parametric testing revealed significant variation in contig counts across tribes (Kruskal-Wallis χ²₃ = 6.59, p = 0.09), with post-hoc tests showing Orthopodmyiini produced significantly more contigs than Aedini (Dunn’s, p = 0.048). Contig lengths also differed by tribe (ANOVA: F₃,_17_ = 2.63, p = 0.084), though pairwise comparisons did not survive multiple-testing correction (Tukey HSD, p > 0.05).

**Fig 1:**
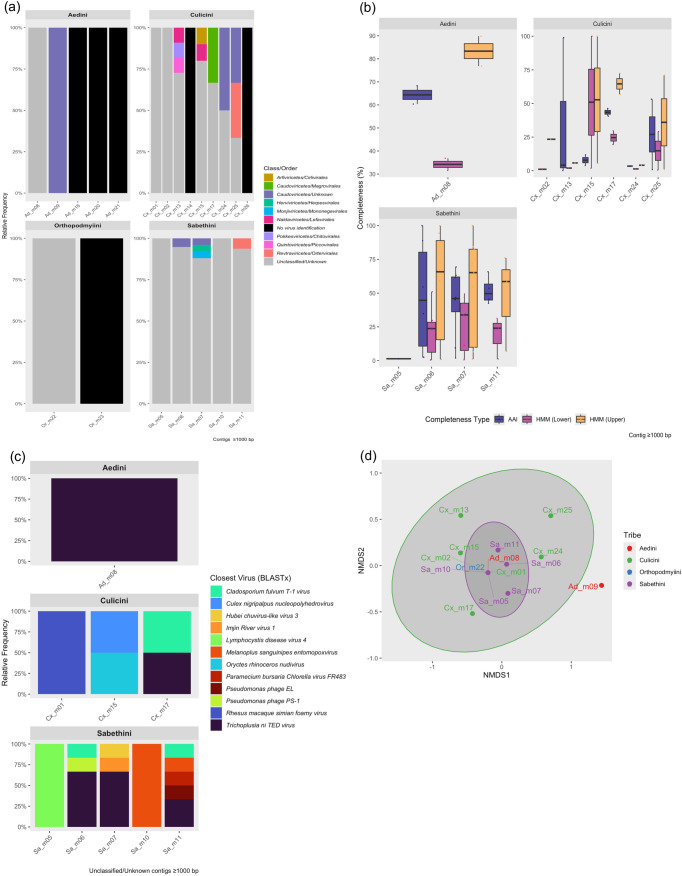
Viral community structure and diversity in non-urban mosquitoes from Colombia. **(a)** Relative abundance of classified viral families in mosquito viromes based on contigs ≥1000 bp, grouped by mosquito tribe (Aedini, Culicini, Orthopodmyiini, Sabethini). **(b)** Viral genome completeness scores by sample and tribe for different estimation methods (AAI, HMM lower, HMM upper), for contigs ≥1000 bp. **(c)** Closest viral relatives of unclassified/unknown contigs, as determined by BLASTx. **(d)** Non-metric multidimensional scaling (NMDS) of virome community composition based on Bray–Curtis dissimilarity of Hellinger-transformed abundance data. Ellipses indicate group centroids and 95% confidence intervals.

Analysis of assembled contigs ≥1000 bp with geNomad revealed that unclassified and unknown viral sequences predominated in all mosquito samples. This pattern of unclassified viral dominance was consistent across most mosquito tribes and samples (Fig 1a, S2 Table**).** The majority of contigs in Aedini, Orthopodmyiini, and Sabethini were either unclassified or showed no viral identification. In contrast, several viral classes and orders were observed among Culicini samples, including Caudoviricetes (Unknown and Magrovirales), Monjiviricetes (Mononegavirales), Quintoviricetes (Piccovirales), Naldaviricetes (Lefavirales), Revtraviricetes (Ortervirales), and Pokkesviricetes (Chitovirales) (Fig 1a, S2 Table). These findings underscore the high proportion of novel or highly divergent viral sequences present in wild mosquito populations, as well as the marked variability in virome composition among different mosquito tribes and samples.

CheckV analysis of assembled viral contigs (≥1000 bp) revealed highly variable genome completeness across mosquito tribes and samples (Fig 1b). In Aedini, contigs from sample Ad_m08 exhibited moderate completeness, with AAI-based estimates clustering around 64%, while sample Ad_m09 yielded only a single contig with insufficient data for assessment (Fig 1b). Among Sabethini, samples Sa_m06, Sa_m07, and Sa_m11 showed the broadest range of completeness values, with both AAI and HMM-derived estimates spanning from low to near-complete (>80%), indicating a mixture of partial and high-quality viral genomes (Fig 1b). In contrast, several Sabethini (Sa_m05, Sa_m10) and Orthopodmyiini (Or_m22) samples yielded only single contigs with very low or unassessed completeness (Fig 1b). For Culicini, completeness estimates varied widely among samples: Cx_m13, Cx_m15, and Cx_m17 included contigs with both low and high HMM-based completeness, whereas Cx_m02, Cx_m24, and Cx_m25 were predominantly associated with lower completeness scores (Fig 1b). Notably, many contigs, especially in Cx_m01, lacked sufficient similarity to reference genomes to yield completeness estimates, underscoring the novelty of the detected sequences.

To further characterize unclassified or unknown viral contigs (≥1000 bp), BLASTx searches were performed against the NCBI viral protein database to identify the closest known viral relatives (Fig 1c, S2 Table**).** The majority of contigs in Aedini (Ad_m08) were most similar to Trichoplusia ni TED virus, suggesting a high prevalence of related viral elements in this sample. In Culicini, distinct viral signatures were observed across samples: Rhesus macaque simian foamy virus (Cx_m01), Culex nigripalpus nucleopolyhedrovirus and Oryctes rhinoceros nudivirus (Cx_m15), and Cladosporium fulvum T-1 virus and Trichoplusia ni TED virus (Cx_m17) represented the predominant closest matches. Sabethini samples displayed the highest viral diversity, with contigs matching a variety of eukaryotic and prokaryotic viruses, including Lymphocystis disease virus 4, Cladosporium fulvum T-1 virus, Pseudomonas phage PS-1, Trichoplusia ni TED virus, Hubei chuvirus-like virus 3, Imjin River virus 1, Melanoplus sanguinipes entomopoxvirus, Paramecium bursaria Chlorella virus FR483, and Pseudomonas phage EL. Notably, the presence of both invertebrate and plant-associated viral hits among unclassified contigs highlights the breadth of viral diversity encountered in wild mosquito viromes and the challenges of precise taxonomic assignment in metagenomic studies.

Beta diversity analysis of virome composition among mosquito tribes was performed using Bray–Curtis dissimilarity on Hellinger-transformed abundance data, visualized with NMDS (Fig 1d**).** PERMANOVA (adonis) testing revealed no significant effect of tribe on virome structure (R² = 0.22, F = 1.02, p = 0.382). These results suggest that, within our dataset, virome composition was not primarily driven by mosquito tribe.

### Expanded viral species detection through a complementary, less stringent approach

Screening contigs between 500 and 999 bp with DeepVirFinder, using a stringent cutoff (score > 0.7, p-value < 0.05, q-value < 0.05) to minimize false positives, identified a total of 43,580 putative viral contigs across all samples (range: 2–4,913 per sample; average length: 624 ± 24 bp). All viral contigs identified in this way were subsequently assessed using geNomad’s neural network classifier. Notably, every DeepVirFinder-predicted contig was assigned to the Unclassified/Unknown category, and none could be confidently matched to any known viral class, order or family.

**Fig 2:**
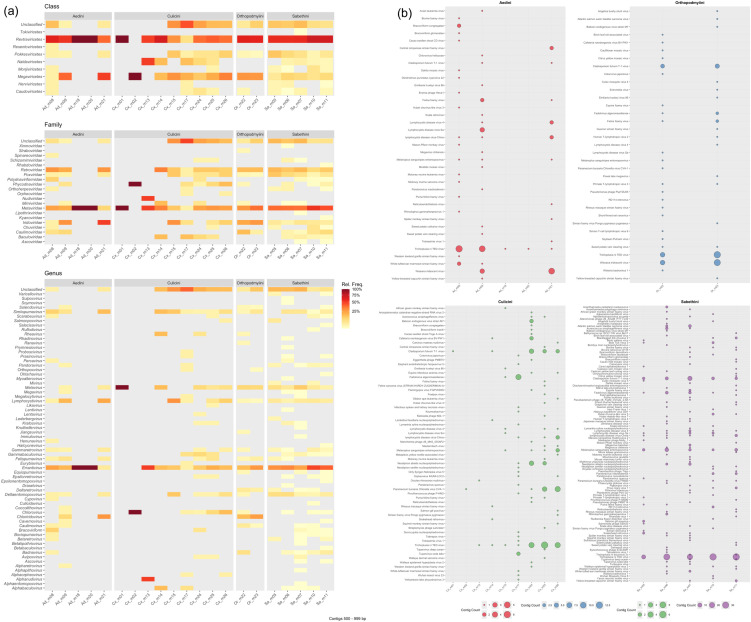
Taxonomic composition and species-level abundance of putative viral contigs (500–999 bp) detected by DeepVirFinder (DVF). **(a)** Relative abundance (frequency) of viral classes, families, and genera across samples and mosquito tribes. **(b)** Absolute abundance (contig count) of each viral species identified in Aedini, Culicini, Orthopodmyiini, and Sabethini pools.

Using BLASTx searches and ICTV-based curation of DeepVirFinder-predicted contigs, we identified 168 viral species, representing the closest BLASTx-assigned relatives, spanning 68 genera and 22 families across all mosquito samples (Fig 2 and S3 Table**).** Virome profiling of mosquito samples from four tribes revealed clear differences in viral composition at the class, family, and genus levels. Revtraviricetes consistently dominated across tribes, particularly in Aedini and Sabethini, while other classes such as Megaviricetes and Pokkesviricetes were more variable and prominent in Culicini (Fig 2a**).** Predominant families included Metaviridae, Retroviridae, Iridoviridae, and Poxviridae, which were present in nearly all samples, while some taxa, such as Nudiviridae, were abundant in specific Culex samples (Fig 2a). At the genus and species levels, viral community composition was highly variable, with certain taxa such prevalent in specific tribes and many species detected sporadically or uniquely (Fig 2a).

In the five Aedini pools, we detected 36 viral species, with Trichoplusia ni TED virus (TniTedV) remaining the most abundant in nearly all Aedini samples, consistent with earlier findings. Other frequently detected viruses included Melanoplus sanguinipes entomopoxvirus (MSEV) and Lymphocystis disease virus Sa (LCDV-Sa). As previously noted, a lower overall viral diversity was apparent in *Aedes albopictus* compared to other Aedini pools, likely reflecting reduced sequencing depth in these samples (Fig 2b). Within the *Culex* (Culicini) samples, TniTedV was also highly prevalent, alongside Cladosporium fulvum T-1 virus (CfuT1V), LCDV-Sa, and Oryctes rhinoceros nudivirus (OrNV). Notably, several viruses of vertebrate hosts were identified, such as Equine infectious anemia virus (EIAV) and Walleye epidermal hyperplasia virus 2 (WEHV2). Sample Cx_m13 was unique in containing the species-specific Culex nigripalpus nucleopolyhedrovirus (CuniNPV) (Fig 2b). For the two Orthopodmyiini samples, we identified 26 viral species, with TniTedV, LCDV-Sa, MSEV, CfuT1V, and Equine foamy virus (EFVeca) comprising the most abundant taxa (Fig 2b). In the Sabethini tribe, the highest species richness was observed, with 110 viral species detected across five samples. Here, most species corresponded to insect-specific viruses, but several vertebrate-associated retroviruses, including Atlantic salmon swim bladder sarcoma virus, EIAV, and Yellow-breasted capuchin simian foamy virus (SFVocr), were also present (Fig 2b). Across Culicinae tribes, TniTedV, LCDV-Sa, MSEV, and CfuT1V consistently emerged as the most abundant viruses, highlighting the recurring presence of certain viral taxa across distinct mosquito lineages.

### Virome diversity

**Fig 3:**
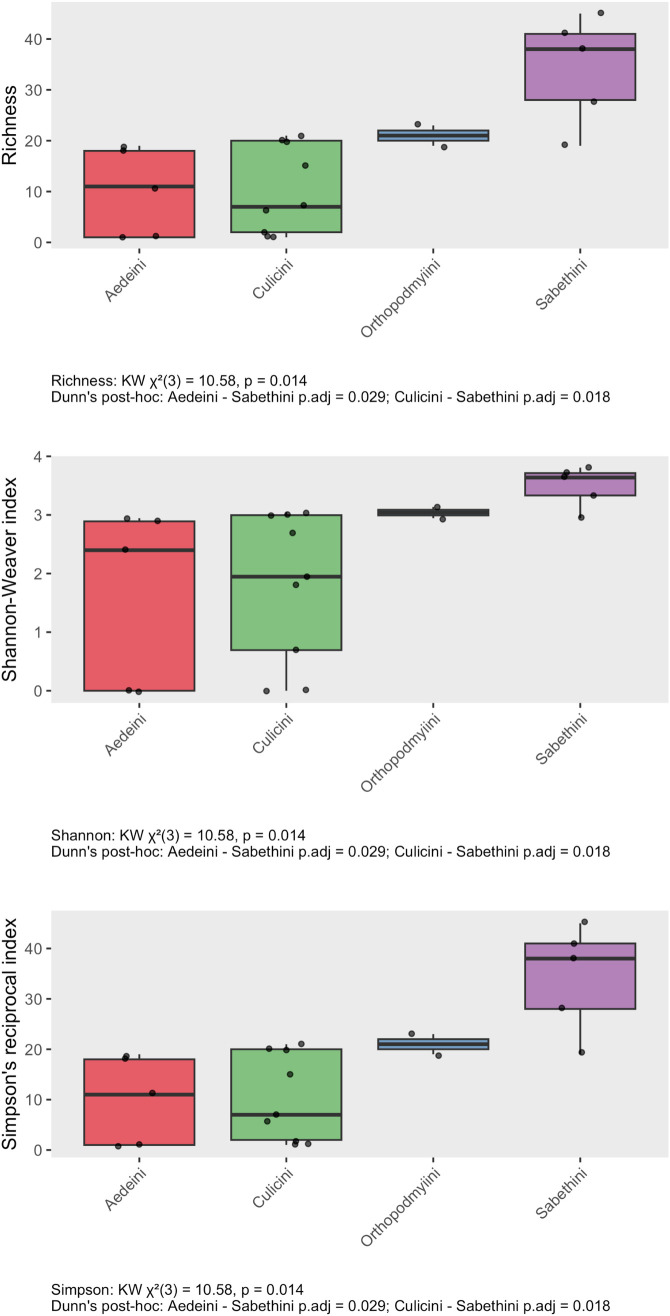
Virome Alpha diversity in non-urban mosquitoes of Colombia. Richness, Shannon-Weaver’s and Simpson’s reciprocal indexes based on 168 virus species identified in 21 mosquito samples of Tribes Aedini, Culicini, Orthopodmyiini, and Sabethini.

Based on the total of 168 virus species identified across all samples using the DeepVirFinder method, viral alpha diversity indices revealed clear differences among mosquito tribes (Fig 3). Sabethini samples exhibited the highest species richness (median = 38), as well as the highest values for both Shannon-Weaver (median = 3.7) and Simpson’s reciprocal index (median = 38), indicating substantial virome richness and evenness. Orthopodmyiini samples also showed elevated diversity across all indices (median richness = 21, Shannon = 3.0, Simpson = 21). In contrast, Aedeini and Culicini samples generally displayed lower richness and diversity, with several samples containing only a single viral species and much lower diversity index values (Aedeini median richness = 11; Culicini median richness = 7). Statistical analysis confirmed that differences in richness, Shannon, and Simpson’s indices among tribes were significant (Kruskal–Wallis test, χ²(3) = 10.58, p = 0.014 for all three metrics). Dunn’s post-hoc tests indicated that Sabethini samples were significantly more diverse than both Aedeini (p.adj = 0.029) and Culicini (p.adj = 0.018) across all diversity indices. No significant pairwise differences were observed between Orthopodmyiini and the other tribes (Fig 3).

## Discussion

### Culicinae mosquitoes in Colombia: Diversity and public health impact

Accurate identification of Culicinae species is essential for understanding their distribution, ecology, and potential role in pathogen transmission. Despite using standard taxonomic keys and expert personnel, species identification was challenging due to overlapping morphological traits and cryptic species. Morphological analysis confirmed the presence of *Aedes albopictus*, *Ochlerotatus* sp., *Culex* sp., *Orthopodomyia* sp., *Wyeomyia* sp., *Sabethes* sp., and *Trichoprosopon* sp., but limitations persisted. Molecular techniques like DNA barcoding and phylogenetic analysis offer greater precision, uncovering hidden diversity within morphologically similar taxa. We are currently implementing an integrative taxonomic approach combining morphology and molecular data for more accurate species classification.

Colombia’s biodiversity includes at least 324 mosquito species, many from the Culicinae subfamily [22]. Culicinae mosquitoes thrive in diverse environments, from tree holes and bamboo stumps to artificial containers like discarded tires and water storage tanks. In our study, we collected 147 adult mosquitoes from two distinct ecosystems. In the Río Claro River basin, a protected natural area, we found a diverse range of species, including *Ochlerotatus* sp., *Wyeomyia* sp., *Sabethes* sp., *Trichoprosopon* sp., and *Culex* sp., illustrating their ability to persist in undisturbed environments. Meanwhile, in Santa Fe de Antioquia, a semi-rural area, species such as *Aedes albopictus*, *Aedes* sp., *Orthopodomyia* sp., and *Culex* sp. were identified, demonstrating their adaptability to human-modified landscapes. This ecological flexibility complicates vector control, requiring habitat-specific strategies.

Culicinae mosquitoes pose significant public health challenges in Colombia due to their ability to transmit arboviruses such as dengue virus (DENV), yellow fever virus, Zika virus (ZIKV), O’nyong-nyong, and West Nile virus [1–5]. Urbanization and climate change are expanding their geographic range, while deforestation and human encroachment into forested areas increase contact between humans and sylvatic mosquito species. In our study, we identified *Ae. albopictus* in Santa Fe de Antioquia, reinforcing its relevance in disease transmission. Additionally, the presence of *Orthopodomyia* sp. and *Wyeomyia* sp. highlights the need for further research into their potential vector roles. Traditional control methods may not be equally effective across all habitats, necessitating integrated vector management (IVM) strategies that combine environmental modification, biological control, and community engagement. Region-specific approaches informed by ecological and epidemiological data will be crucial for mitigating the risks posed by Culicinae mosquitoes.

### Virome composition and diversity in Culicinae Mosquitoes in Colombia

Despite increasing interest in the virome composition and diversity in mosquitoes, information for Neotropical sylvatic or no-urban species is very scarce, particularly for mosquito species harbouring arboviruses and those potentially involved in zoonotic or phytonotic diseases. Here we analyzed the virome through metagenome analysis in 147 field-caught mosquitoes of one species (*Ae. albopictus*) and 19 morphotypes belonging to the genera *Culex* sp., *Aedes* sp., *Ochlerotatus* sp., *Trichoprosopon* sp., *Wyeomyia* sp., and *Orthopodomyia* sp., representing the tribes Aedini, Culicini, Orthopodmyiini, and Sabethini. The importance of endogenous viruses, as reported in other mosquitoes, underscores the need to investigate their potential roles, as they may significantly influence host physiology, immune responses, and ecological interactions in the evolution of the mosquito microbiome [50,51].

The diversity in virome composition in sylvatic mosquito species and populations has been described recently, and differences may depend on the host ecology as well as environmental and climatic conditions [8]. Here we performed a hybrid approach to maximize both the sensitivity and specificity of viral detection. Comparison of the two approaches revealed some overlap as well as differences in the most abundant viral species detected. Using the high-confidence geNomad pipeline and BLASTx annotation of previously unclassified contigs, the most frequently detected and abundant viruses across samples included Trichoplusia ni TED virus, Cladosporium fulvum T-1 virus, Lymphocystis disease virus 4, Oryctes rhinoceros nudivirus, and Melanoplus sanguinipes entomopoxvirus. With the less stringent DeepVirFinder approach combined with BLASTx, a broader range of viral species was detected, but the most prevalent and abundant species were again Trichoplusia ni TED virus, Lymphocystis disease virus Sa, Cladosporium fulvum T-1 virus, Equine foamy virus, and Oryctes rhinoceros nudivirus. Both methods consistently recovered insect-specific viruses as the dominant taxa, although the DVF approach also identified a greater diversity of rare and potentially novel viruses, including several large DNA viruses and vertebrate-associated retroviruses in lower abundance. This overlap in core viral taxa across methods underscores the robustness of these identifications, while the expanded diversity detected with DVF highlights the added sensitivity of less stringent detection approaches in characterizing the mosquito virome.

For DVF-based approach, we detected 168 viral species from 68 genera and 22 families across 21 pools of mosquitoes, with viruses belonging to the *Metaviridae, Retroviridae, Iridoviridae,* and *Poxviridae* being especially abundant, excepting for *Ae. albopictus* samples and some *Culex* sp. that showed an extremely low number of viral sequences, with low number of assembled contigs obtained, that could be due to low viral content in the sample or low technical recovery after sequencing and processing. The virome found here harbored all virus families reported in Colombia so far (S4 Table). The highest viral diversity was observed in Sabethini samples, which is consistent with previous findings of high genetic diversity within this tribe [18]. Orthopodmyiini samples also exhibited elevated diversity across all indices; notably, this is a novel observation, as there is little prior information regarding the virome composition of this tribe. In contrast, Aedeini and Culicini samples generally showed lower richness and diversity, with several samples containing only a single viral species and much lower diversity index values. A substantial proportion of viral sequences could not be confidently assigned to known taxa, highlighting the fact that the taxonomic knowledge of insect-specific viruses (ISVs) and sylvatic mosquito-associated viruses (SSVs) in non-urban environments remains largely undescribed and underrepresented in current reference databases.

A recent study on *Anopheles darlingi* from the Northern Andean valleys of Colombia revealed a diverse set of RNA viral sequences, with homology to insect-specific viruses from families such as Rhabdoviridae, Partitiviridae, Metaviridae, Tymoviridae, Phasmaviridae, and Totiviridae, as well as the orders Ortervirales and Riboviria [7]. Notably, the virome composition of *An. darlingi* was stable across regions, with a core group of viral operational taxonomic units (vOTUs) shared among populations. Similarly, our results uncovered a broad diversity of both RNA and DNA insect-specific viruses, including Rhabdoviridae, Metaviridae, and Totiviridae, alongside several additional viral families and orders. Core viral taxa such as Trichoplusia ni TED virus, Cladosporium fulvum T-1 virus, and Lymphocystis disease virus were consistently detected across multiple mosquito tribes and ecological contexts. This concordance suggests that, despite differences in host taxonomy and environment, a stable set of core ISVs and viral lineages may be widely maintained in sylvatic mosquito populations of the Northern Andes. These findings align with recent global studies reporting high abundances of ISV-containing families such as Flaviviridae, Rhabdoviridae, Iflaviridae, Nodaviridae, Mesoniviridae, Orthomyxoviridae, and Totiviridae, as well as bacteriophages from the Autographiviridae, Drexlerviridae, and Straboviridae families [18]. These results indicate the presence of persistent or “core” virome components in mosquitoes [52,53].

Herein we report for the first time the species-specific virus (SSV) Culex nigripalpus nucleopolyhedrovirus (CuniNPV) which is important for regulating mosquito populations and influencing vector competence, with potential applications as a biocontrol agent [50]. According to previous reports, Trichoplusia ni TED virus (TniTedV) was observed as a dominating virus [18,54–56]. Additionally, several animal and plant-infecting viruses were found in several samples such as Lymphocystis disease virus (LCDV – Pathogen of fish), Melanoplus sanguinipes entomopoxvirus (MSEV – Pathogen of grasshoppers), Oryctes rhinoceros nudivirus (OrNV – Pathogen of beetles), Rhesus macaque simian foamy virus (SFV – infects primates), among others. These results underline the relevance of extensive sequencing surveys to detect sources and vectors of these viruses and to be capable efficiently address challenges by emerging or re-emerging viruses in zoonotic and anthropozoonotic scenarios. The presence of flaviviruses, including both arboviruses and insect-specific flaviviruses, has previously been reported in some mosquito species in a near-disturbed forest of the Magdalena Medio Valley (Hoyos et al., 2021). However, in samples from a distinct geographical location in Colombia analyzed here, we did not observe viral sequences assigned to the Flaviviridae family. This would indicate that at this sampling location and time, there was a very low risk of infection risk with such arboviruses, in agreement with epidemiological surveys at the time of our sampling in this area.

### Unexpected viral identifications and their implications

The DFV-based approach enabled the detection of a wide range of viral sequences, including several unexpected for the region and host species, such as Baboon endogenous virus, Walleye dermal sarcoma virus, Koala retrovirus, and a range of primate, feline, and rodent retroviruses, among others. Notably, we also detected several DNA viruses, despite employing an RNAseq-based protocol. This likely reflects the transcriptional activity of DNA viruses or the presence of DNA viral genomes as persistent particles, but may also result from technical artifacts such as sample carryover or incomplete removal of DNA during library preparation. Although these identifications were based on sequence similarity to entries in public databases, it is important to note that, in some cases, high similarity may be observed over relatively short alignment lengths. Such cases, while passing our quality thresholds, may not provide robust support for true taxonomic assignment. Therefore, the detection of these viruses in Colombian mosquitoes warrants careful and cautious interpretation.

Several factors could account for these findings. Environmental contamination, from imported animals, animal products, or waste, could introduce viral sequences into mosquito habitats. Incidental contact with environmental viral particles is also possible, even though unfed mosquitoes were analyzed. Notably, the reliance on existing reference databases introduces potential biases: novel or highly divergent viruses may be classified as the closest known match, often from unrelated hosts or distant geographic regions.

While we applied stringent quality thresholds and re-examined raw read alignments to minimize false positives, the less stringent DFV approach, by design, increases sensitivity and may also increase the risk of misclassification due to conserved domains, low-complexity regions, or database artifacts. Thus, although our bioinformatics pipeline helps ensure robust detection, some identifications may represent technical or interpretive artifacts. Definitive confirmation would require targeted PCR, Sanger sequencing, or the isolation of complete viral genomes.

These unexpected identifications highlight both the promise and the challenges of metagenomic virome analysis in understudied environments. They emphasize the urgent need to expand reference databases with viral genomes from diverse geographic regions and host taxa, to reduce misclassification and improve the ecological relevance of virome studies. Future research should explore the origins and potential ecological or epidemiological significance of these putatively non-native viruses, considering the possibility of environmental contamination, introductions via non-native hosts, or the discovery of novel, uncharacterized viral lineages.

## Conclusions

This study presents the first comprehensive analysis of the virome in Neotropical sylvatic mosquitoes from the Northern Andean Valleys of Colombia, offering insights into Culicinae mosquitoes and their potential roles in hosting predominant insect-specific viruses (ISVs) and other animal and plant pathogens. The mosquitoes, classified within the Tribes Aedini, Culicini, Orthopodmyiini, and Sabethini, showed considerable variation in virome composition, particularly among low-frequency ISVs, suggesting that virome diversity is shaped by mosquito species, environment, and lifestyle. The detection of species-specific viruses like Culex nigripalpus nucleopolyhedrovirus (CuniNPV) highlights potential future applications in mosquito population control. The absence of flaviviruses suggests a minimal risk of arbovirus transmission at the time and location of sampling. This study provides a baseline for future virome research in sylvatic Culicinae mosquitoes in Colombia and beyond, and highlights the need for further investigation into the relationships between the endogenous virome and associated bacterial communities in mosquito biology and pathogen transmission.

## Supporting information

S1 Table Summary of read counts, assembly statistics, and mean contig lengths (bp) for virus taxonomic identification in mosquitoes in Colombia. (XLSX)

S2 Table Summary for viral taxonomy contigs counts identified in 21 pools of non-urban mosquitoes of Tribes Aedini, Culicini, Orthopodmyiini, and Sabethini using more-confident approach in contigs ≥1000 bp with geNomad. **(a)** Number of classified viral contigs (≥1000 bp) detected in each mosquito virome sample, grouped by viral taxonomic class or family and sample code (organized by mosquito tribe: Aedini, Culicini, Orthopodmyiini, and Sabethini). **(b)** Number of unclassified or unknown contigs (≥1000 bp) in each sample with the indicated viral species as their closest match based on BLASTx analysis. N.d. indicates not detected.
(XLSX)

S3 Table Absolute abundance of viral species identified in mosquito viromes from 500–999 bp contigs using the DeepVirFinder-based hybrid pipeline for 21 pools of non-urban mosquitoes of Tribes Aedini, Culicini, Orthopodmyiini, and Sabethini. (XLSX)

S4 Table Summary of virus species reported to Colombia so far. (XLSX)
